# Frationation of hydrolysate from corn germ protein by ultrafiltration: In vitro antidiabetic and antioxidant activity

**DOI:** 10.1002/fsn3.1529

**Published:** 2020-04-05

**Authors:** Amin Karimi, Mohammad Hossein Azizi, Hassan Ahmadi Gavlighi

**Affiliations:** ^1^ Department of Food Science and Technology Faculty of Agriculture Tarbiat Modares University Tehran Iran

**Keywords:** antidiabetic potential, antioxidant, defatted corn germ, protein hydrolysates

## Abstract

In the present work, defatted corn germ was hydrolyzed by three proteases and further separated by sequential ultrafiltration with different molecular weight cutoff (100, 10, 2 kDa). Corn germ protein hydrolysate (CGPH) and their fractions were investigated for antioxidant activity, α‐glucosidase, α‐amylase, and DPP‐IV inhibitory activity. The degree of hydrolysis (DH) after 2 hr was 17.5%, 11.14%, and 2.05% for alcalase, trypsin, and flavourzyme, respectively. Trypsin hydrolysate showed the highest DPPH and ABTS^+^ radical scavenging and Fe^2+^ chelating activity, but a lower α‐glucosidase inhibitory activity. F1 fraction (<2 kDa) exhibited highest radical scavenging and α‐glucosidase inhibitory activity. While F2 fraction (2–10 kDa) showed the higher Fe^2+^ chelating and α‐amylase inhibitory activity, F1 fraction of flavourzyme showed the highest α‐glucosidase inhibitory and F2 fraction of alcalase and flavourzyme exhibited highest α‐amylase inhibitory activity. Hydrolysate and F1 fraction of alcalase and F2 fraction of trypsin showed the highest DPP‐IV inhibitory activity. RP‐HPLC results showed that trypsin hydrolysate had higher levels of high‐hydrophobic peptides. The amino acid composition of the F1 fractions showed high levels of hydrophobic amino acids. Thus, CGPHs may be used as a potential source of antioxidant and antidiabetic peptides in food industry and pharmaceutical application.

## Graphical abstract

Corn germ protein hydrolysate (CGPH) by three proteases and their fractions were investigated for antioxidant activity, α‐glucosidase, α‐amylase, and DPP‐IV inhibitory activity. F1 fraction (<2 kDa) exhibited highest radical scavenging and α‐glucosidase inhibitory activity. Hydrolysate and F1 fraction of alcalase and F2 fraction of trypsin showed the highest DPP‐IV inhibitory activity.

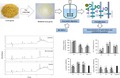

## 1 INTRODUCTION

In view of food industry and human health, prevention of lipid oxidation and generation of free radicals in the foodstuff and living body tissue are important for control of quality of food and diseases (Zhang et al., 2016, 2019). Antioxidants are often used in food formulation to eliminate the effects of free radicals and to inhibit lipid oxidation (Zhang et al., 2016). The use of synthetic antioxidants (BHA and BHT) has serious limitations due to the potential toxic effects on human health. Therefore, many studies are necessary on production and application of antioxidants from natural sources without side effects on human health (Jin, Liu, Zheng, Wang, & He, 2016; Nasri, 2017).

Diabetes mellitus is serious metabolic disease and characterized by high level of blood glucose (Bhandari, Jong‐Anurakkun, Hong, & Kawabata, 2008; Vilcacundo, Martínez‐Villaluenga, & Hernández‐Ledesma, 2017). One approach in the management of diabetes is slowing down the absorption of glucose through the inhibition of α‐glucosidase and α‐amylase in hydrolysis of carbohydrates (Alu'datt et al., 2012; Bhandari et al., 2008; Connolly, Piggott, & FitzGerald, 2014). α‐Amylase and α‐glucosidase inhibitors can inhibit both enzymes activities and decrease rate of digestion of starch and disaccharides to glucose, and as results, less glucose is absorbed in the small intestine (Vilcacundo et al., 2017). Another approaches for control of diabetes are through inhibition of dipeptidyl peptidase‐IV (DPP‐IV) activity that increases the half‐life of total circulating GLP‐1 (peptides that stimulates glucose‐dependent insulin secretion in pancreatic β‐cells) by preventing it from degradation and inactivation. Thereafter, prolonged secretion of insulin resulted in more absorption of glucose by the tissues and decreased in plasma glucose (Estrada‐Salas, Montero‐Morán, Martínez‐Cuevas, González, & Barba de la Rosa, 2014; Vilcacundo et al., 2017; Zambrowicz et al., 2015). Hence, in the past few years, enzyme inhibitors have developed for control of the type 2 diabetes. In this concept similar to α‐amylase and α‐glucosidase inhibitor, concern about synthetic drugs and toxicity and side effect should be noticed and using of natural and safe inhibitors as antidiabetic agents is considered (Wang et al., 2019).

Other than nutritional properties, protein hydrolysates and biopeptides have various biological activities such as antioxidant, antibacterial, antihypertensive, and antidiabetic potential depending on amino acid composition, sequencing, hydrophobicity, and chain length (Nasri, 2017). In recent years, various food protein hydrolysates have been reported to be antioxidant and antidiabetic potential in barley (Alu'datt et al., 2012), *Palmaria palmata* (Harnedy & FitzGerald, 2013), egg yolk protein (Zambrowicz et al., 2015), pinto beans (Ngoh & Gan, 2016), and cumin seeds (Siow & Gan, 2016).

Defatted corn germ (DCG) is by‐product of the corn oil industry. Its goes mainly into animal feed, and very low amount has been used as ingredient in food formulations (Barbieri & Casiraghi, 1983). Production of hydrolysate from corn germ and evaluation of antihypertensive activity has been studied (Parris, Moreau, Johnston, Dickey, & Aluko, 2008). However, limited information is available on antioxidant and antidiabetic properties of DCG hydrolysate. So, in this study, the ability of three proteases (alcalase, flavourzyme, trypsin) to generation hydrolysate from corn germ protein was evaluated and then antioxidant activity and DPP‐IV, α‐amylase and α‐glucosidase inhibitory activity of hydrolysates, and their fractions were investigated.

## 2 MATERIALS AND METHODS

### 2.1 Materials

Corn germ was obtained from Glucosan Ind Co. DPPH (2,2‐Diphenyl‐1‐picrylhydrazyl), ABTS (2,2′‐azino‐bis (3‐ethylbenzthiazoline‐6‐sulfonic acid) diammonium salt)), ferrozine (3‐(2‐Pyridyl)‐5,6‐diphenyl‐1,2,4‐triazine‐4′,4′′‐disulfonic acid sodium salt), PNPG (4‐nitrophenyl α‐d‐glucopyranoside), PAHBAH (4‐hydroxybenzhydrazide), trolox (6‐hydroxy‐2,5,7,8‐tetramethylchroman‐2‐carboxylic acid), 1,10‐phenanthroline, acarbose, chromogenic substrate Gly‐Pro‐p‐nitroaniline (Cat no. G0513) were purchased from Sigma‐Aldrich company. l‐Histidine (Cat no. 104351) was purchased from Merck company. Diprotin A (Ile‐Pro‐Ile) was purchased from Cayman chemical company. All other chemicals were of analytical grade.

Enzymes: Alcalase 2.4 L (Protease from *Bacillus licheniformis*), trypsin from porcine pancreas (Cat no. T4799), porcine pancreatic α‐amylase (Cat no. A3176), rat intestinal α‐glucosidase (Cat no. I1630), and dipeptidyl peptidase‐IV (DPP‐IV) (Cat no. 317640‐M) are obtained from Sigma‐Aldrich. Flavourzyme 500 MG from *Aspergillus oryzae* (Cat no. P6110) was purchased from Novozymes company.

### 2.2 Preparation of corn germ protein hydrolysate (CGPH)

Ground samples of corn germ were defatted with n‐hexane using soxhlet apparatus and dried at room temperature overnight. The dried material was ground and sieved through a 0.4 mm sieve (Mesh no. 40). The DCG was hydrolyzed according to the method of He, Girgih, Malomo, and Aluko (2013) with alcalase, flavourzyme, and trypsin enzymes. DCG suspension (5% w/v) was heated to the appropriate temperature and pH of each enzyme (alcalase pH 8 at 50°C, flavourzyme pH 7 at 50°C, and trypsin pH 7 at 50°C), and enzymes were added based on protein content of DCG at 1:20 ratio. The reaction continued with pH‐stat method for 2 hr, and then enzymes were inactivated by heating at 95°C for 15 min and finally, samples centrifuged at 10,000 *g* for 15 min and supernatant was freeze‐dried as CGPH.

### 2.3 Degree of hydrolysis (DH)

The pH‐stat method of Adler‐Nissen (1986) was used for measuring and calculating degree of hydrolysis (DH).

$$\text{DH}\left(\%\right)=B\times N_{b}/\left(\alpha \times M_{p}\times h_{\mathrm{tot}}\right)\times 100$$

The *h_tot_* of this equation was 7.75 meq/g (Zhang, Pang, & Xu, 2011).

### 2.4 Reversed‐phase chromatography separation of CGPH

The hydrophobicity of peptides from CGPHs was determined using an Azura HPLC system (Knauer). CGPHs were dispersed (10 mg/ml) and filtered through 0.2 μm cellulose acetate filters. RP‐HPLC was carried out following the procedure reported by Connolly et al. (2014) with some modifications. In brief, the samples were separated on Eurosil Bioselect column (250 × 4.6 mm ID, 5 μm particle size, 300 Å pore size) using solvent A (0.1% (v/v) trifluoroacetic acid (TFA) in water) and solvent B (0.1% (v/v) TFA in acetonitrile) under gradient conditions. The column was equilibrated using 100% A solvent. Elutions were performed as follows: 0–30 min, 0%–60% B; 30–35 min, 60% B; 35–45 min, 60%–10% B, and 45–50 min, 10% B. The UV‐Vis photodiode‐array detector (DAD 2. one langmuir, Knauer) was set at 214 nm for measuring absorbance.

### 2.5 Fractionation of hydrolysates

CGPH was fractionated by the ultrafiltration membranes with the molecular weight cutoff (MW) of 100, 10, and 2 kDa (Sartorius ‐VivaFlow 200) subsequently and namely F1, F2, F3, and F4. F1 corresponds to peptide with molecular weight lower than 2 kDa, F2 to peptide with MW between 2 and 10 kDa, F3 to peptides with MW between 10 and 100 kDa and F4 to undigested proteins and other compounds fragments with MW more than 100 kDa. All fractions were lyophilized and stored at −20°C for using all analysis.

### 2.6 Determination of antioxidant activities

#### 2.6.1 DPPH radical scavenging activity

The DPPH radical scavenging effect of CGPH and fractions were measured according to the method of Zheng et al. (2015). The DPPH solution (500 μl) of each samples (0.5 mg/ml) was added to 500 μl DPPH in methanol (0.1 mM) and left for 30 min in dark place at 25°C. The ability of samples to scavenge of DPPH free radicals was measured at 517 nm with the UV–visible spectrophotometer (Agilent‐Carry 60). Trolox concentrations ranging from 20 to 200 µmol/L were used for calculation of scavenging activity of the hydrolysates and results expressed as µmol trolox/g dry matter of samples.

#### 2.6.2 ABTS^+^ radical scavenging activity

The scavenging effect of CGPH and fractions on ABTS^+^ radical were performed according to the method of Ngoh and Gan (2016). The ABTS solution (980 µl) was added to 20 µl of samples (2.5 mg/ml) and mixed vigorously and then incubated in the dark at 25°C for 10 min and absorbance was measured at 734 nm. The result was expressed as µmol trolox equivalents (concentrations ranging from 50 to 1,100 µmol/L) per g dry matter of samples.

#### 2.6.3 Hydroxyl radical scavenging activity

The hydroxyl radical scavenging effect of CGPH and fractions were measured following the method of Wang et al. (2014). Briefly, 200 μl samples (5 mg/ml), 100 μl FeSO_4_ (1.865 mM), and 100 μl of 1,10‐phenanthroline solution (1.865 mM) were mixed thoroughly and were allowed to stand for 10 min and then 100 μl of H_2_O_2_ (0.03% v/v) was added to the mixture. The mixture was then incubated at 37°C for 60 min, and after that, the absorbance was measured at 536 nm. Histidine was used as a standard curve (300–4,000 μmol/L). The result was expressed as µmol of histidine equivalents/g dry matter of samples.

#### 2.6.4 Ferrous ion chelating activity

Chelation of Fe^2+^ ions by hydrolysates and fractions was estimated by the method of Sarteshnizi, Sahari, Gavlighi, Regenstein, and Nikoo (2019). The sample solutions of 5 mg/ml hydrolysate and fractions (500 μl) were mixed with 1,850 μl H_2_O and 50 μl FeCl_2_ (2 mM). After 3 min, 100 μl of 5 mM ferrozine aqueous solution was added and the mixture was allowed to react for 20 min. The absorbance of complex was measured at 562 nm. A standard curve was obtained by using EDTA (20–250 μmol/L). The result was expressed as µmol EDTA equivalents/g dry matter of samples.

#### 2.6.5 α‐**Glucosidase inhibition assay**

Inhibition of α‐glucosidase (rat intestinal) by CGPH hydrolysates and fractions were measured using the method of Connolly et al. (2014) with some modifications. After extraction of the enzyme from rat intestinal acetone powders, the resulting solution was diluted to 90 mU/ml. One hundred microliter of sample solution (20 mg/ml) was mixed with 200 μl of α‐glucosidase and incubated at 37°C for 10 min. After preincubation, 5 mM PNPG solution (100 μl) was added and incubated at 37°C for 30 min and the absorbance of the solution was scanned every 2 min at 405 nm. The phosphate buffer was used as a control instead of sample solution. The IC_50_ value of acarbose was used as the positive control. The following equation was used to assess percentage inhibition of α‐glucosidase activity:

$$\text{inhibition of}\;\alpha \;-\;\text{glucosidase}\;\left(\%\right)=\left[\text{Ac}-\text{As}/\text{Ac}\right]\times 100$$

where As and Ac represent the slope of curve for absorbance of samples and control, respectively.

#### 2.6.6 α**‐Amylase inhibition assay**

α‐Amylase inhibition was determined according to the method of Alu'datt et al. (2012) with some modifications. Briefly, 100 μl of sample solution (10 mg/ml) and 100 μl of α‐amylase solution (0.5 U/ml) were incubated at 37°C for 5 min. After preincubation, 100 μl of 0.5% (w/v) starch solution was added. Then, the reaction mixture was incubated for 20 min at 37°C. In the following, reaction mixture was heated at 100°C for 10 min and then cooled down to room temperature and centrifuged for 2 min at 16060 *g* to separate the undigested starch. Twenty microliters of supernatant was mixed with 1 ml of PAHBAH and heated to 70°C for 10 min. Finally, solution was cooled at room temperature and absorbance was measured at 410 nm. The IC_50_ value of acarbose was used as the positive control. The following equation was used to assess percentage inhibition of α‐amylase activity:

$$\text{inhibition of}\;\alpha \;-\;\text{amylase}\;\left(\%\right)=\left[1-\left(\text{As}-\text{Ab}\right)/\text{Ac}\right]\times 100$$

where As, Ab, and Ac represent the absorbance of sample, blank (phosphate buffer, enzyme, sample), and control (starch, buffer, enzyme), respectively.

#### 2.6.7 Dipeptidyl peptidase‐IV (DPP‐IV) inhibition assay

DPP‐IV inhibition was determined following the procedure reported by Nongonierma and FitzGerald (2013). Briefly, 25 μl sample solution (5 mg/ml) was mixed by 25 μl of Gly‐Pro‐pNA, as substrate (0.2 mM) and incubated for 10 min at 37°C. The reaction was started by the addition of DPP‐IV (final concentration 0.0025 units/ml) for 1 hr at 37°C and the absorbance of *p*‐nitroaniline released was read at 405 nm. The IC_50_ value of diprotin A was used as positive control. The inhibitory activity of sample on DPP‐IV was calculated by the following equation:

$$\text{Inhibition of DPP}\;-\;\text{IV}\;\left(\%\right)=\left[\left(\text{Ac}-\text{Acb}\right)-\left(\text{As}-\text{Asb}\right)/\left(\text{Ac}-\text{Acb}\right)\right]\times 100$$

where As, Asb, Ac, and Acb represent the absorbance of sample, sample blank (sample, buffer, substrate), control (enzyme, substrate, buffer), and control blank (buffer, substrate), respectively.

### 2.7 Amino acid composition

Samples were hydrolyzed with 6 M HCl at 110°C for 24 hr. Subsequently, the digested samples were lyophilized. Amino acid profile of the samples was determined using an OPA method by HPLC (Knauer), RP‐C18 Hypersil ODS column (250 × 4.6 mm, particle size 5 µm), and fluorometric detector RF‐530 (Shimadzu‐japan) (Nikoo, Benjakul, Yasemi, Gavlighi, & Xu, 2019).

### 2.8 Statistical analysis

All analyses were carried out in triplicate and data presented as mean ± *SD*. Data analysis was carried out with JMP 10 statistical software using one‐way analysis of variance and Tukey's test to compare differences between samples (*p* < .05).

## 3 RESULTS AND DISCUSSION

### 3.1 Degree of hydrolysis (DH)

**FIGURE 1:**
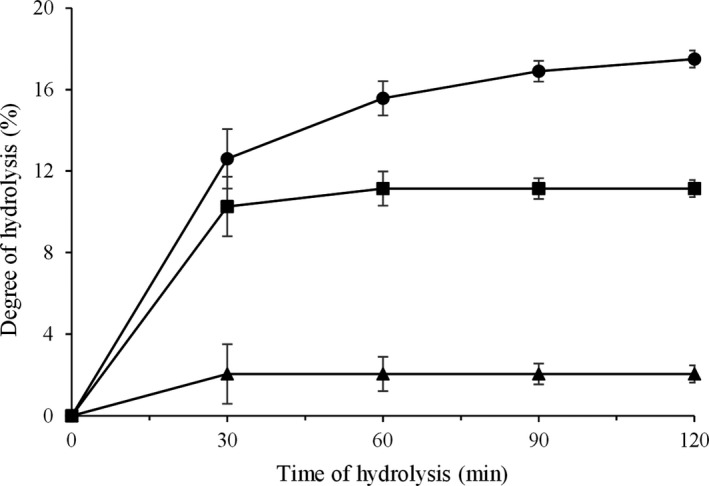
Degree of hydrolysis of corn germ with different enzymes (CGPH with alcalase ●, flavourzyme ▲, trypsin ■). Values represent the average of two independent hydrolysis

DH of all enzymes treated DCG was increased sharply within the initial 30 min of reaction. Thereafter, the rate of DH was decreased (Figure 1). The reduction in hydrolysis rate over time may be related to decreased availability of cleavable peptide bonds within the substrate (Kumar, Chatli, Singh, Mehta, & Kumar, 2016). The highest DH value of hydrolysates was 17.5 ± 0.2%, which was obtained by alcalase, while that of the CGPH produced by flavourzyme was the lowest (2.05 ± 0.4%). Different DH value of each enzyme was due to different cleavage sites of the protease enzymes (Wang et al., 2019). Alcalase is an endo‐peptidase with a broad specificity, which favorably cleaves hydrophobic amino acid (tryptophan, phenylalanine, leucine, isoleucine, valine, and methionine) residues of peptide bonds, and higher DH value can be achieved with longer enzymolysis time. Flavourzyme is an exo‐peptidase that breaks the N‐terminal of peptide chains. While trypsin cleaves exclusively C‐terminal to arginine and lysine (Ambigaipalan, Al‐Khalifa, & Shahidi, 2015; Wang et al., 2019), this result indicates that alcalase is the most efficient for corn germ protein hydrolysis. Similar results were obtained for whey protein (Lin, Tian, Li, Cao, & Jiang, 2012), rapeseed protein (He et al., 2013), camel milk casein (Kumar et al., 2016), hemp seed protein (Ren et al., 2016), quinoa and amaranth proteins (Lina, Omar, Kamal, Kilari, & Maqsood, 2019), and buffalo and bovine caseins (Shazly et al., 2019). DH could be effect on functionality and bioactive properties of hydrolysate, so it is important to control DH in production of new food products (Mudgil, Omar, Kamal, Kilari, & Maqsood, 2019).

### 3.2 Reversed‐phase chromatography separation of CGPH

**FIGURE 2:**
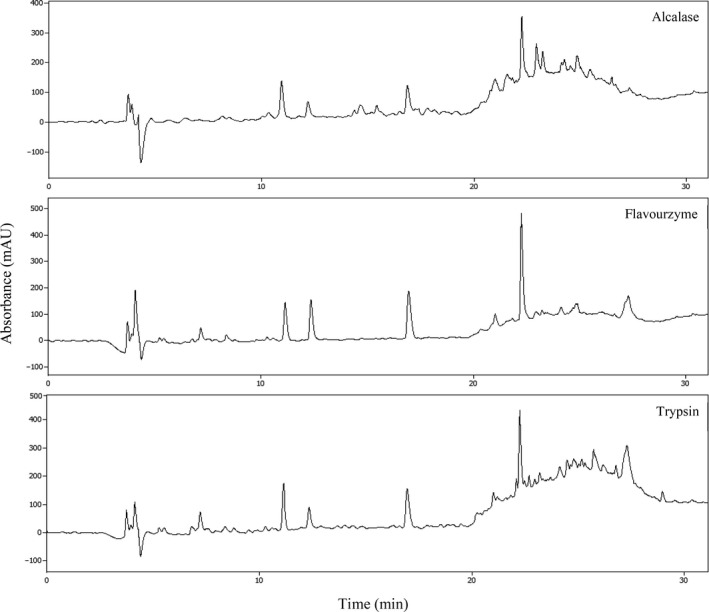
RP‐HPLC of CGPHs with different enzymes (alcalase, flavourzyme, trypsin). Samples (20 μl) were injected and separated on a Knauer 25EK Eurosil Bioselect column (C18, 250 × 4.6 mm, 5 μm)

The ratio of hydrophilic to hydrophobic peptide groups is one of most important parameters that can effect on functional properties. Reversed‐phase HPLC was separated peptides based on the hydrophobic or hydrophilic character that resulted in later elution of hydrophobic peptide compared hydrophilic peptides (Zhang, Mu, & Sun, 2014). The RP‐HPLC profiles of CGPH (Figure 2) by trypsin showed a higher concentration of high‐hydrophobic peptides. Flavourzyme hydrolysate showed a lower concentration of high‐hydrophobic peptides. This effect is probably related to the specific function of each enzyme. The hydrophobicity of hydrolysates and peptides plays an important role in antioxidant activity, (Zambrowicz et al., 2015). An increase in hydrophobicity (As can be seen in Figure 2 for trypsin) will increase their solubility in hydrophobic phase and can improve their interaction with hydrophobic targets, thereby enhancing bioavailability and exhibiting enhanced interaction especially with radical species (Li, Jiang, Zhang, Mu, & Liu, 2008). Moreover, hydrophobic peptides tend to donate protons to reactive radicals and convert free radicals to more stable products and terminate radical chain reaction (Jin et al., 2016; Siow & Gan, 2016).

### 3.3 Antioxidant activities of samples

**FIGURE 3:**
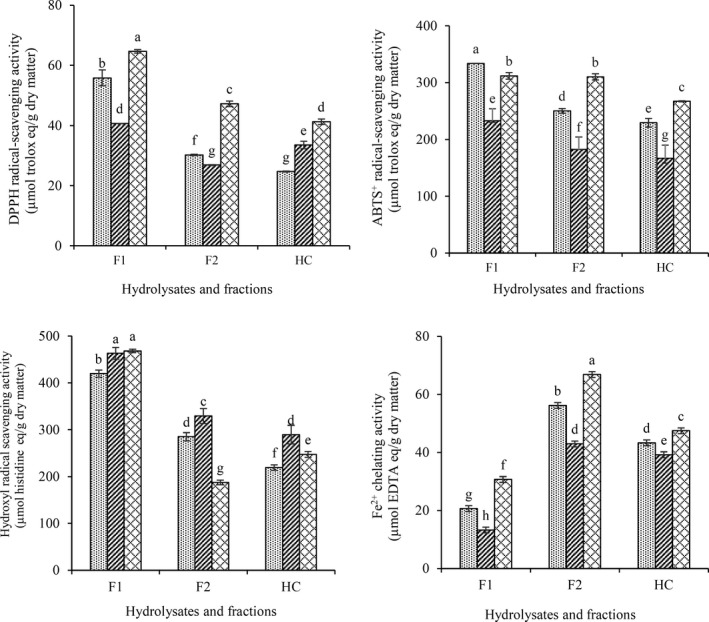
DPPH (a), ABTS^+^ (b), hydroxyl (c) radical scavenging, and Fe^2+^ chelating (d) activity of CGPH obtained from hydrolysis by different enzymes (alcalase 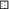, flavourzyme 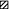, trypsin 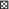) and their ultrafiltration fractions. F1, F2, and HC indicate fractions MW < 2, 2–10 kDa and unfractionated hydrolysate, respectively. Test concentration for DPPH, ABTS^+^, hydroxyl radical scavenging, and Fe^2+^ chelating activity was 0.5, 2.5, 5, and 5 mg/ml respectively. The data marked with different letters are significantly different (*p* < .05)

Due to high turbidity of F3 and F4 fractions and made problem in antioxidant and antidiabetic tests, these fractions were excluded. Antioxidant peptides are new source of natural antioxidant compound that show activity through radical scavenging, metal chelating, hydroperoxide reduction, and inactivating reactive oxygen species (Zambrowicz et al., 2015). The DPPH radical scavenging activity (RSA) of CGPH and fractions showed distinct differences for the hydrolysates obtained by different proteases (Figure 3a). Trypsin hydrolysate was the most active against DPPH radical (64.7 µmol trolox eq/g sample) followed by flavourzyme and alcalase with least activity (*p* < .05). Hydrolysates prepared using trypsin had the highest ABTS^+^ RSA with 267 µmol trolox eq/g sample, while flavourzyme hydrolysates showed the lowest values (167 µmol trolox eq/g sample) (Figure 3b). The similar results were observed by Shazly et al. (2019) on higher ABTS^+^ RSA of buffalo and bovine caseins hydrolysates of trypsin than alcalase, papain, and pepsin. Hydrolysates prepared using flavourzyme showed the highest OH RSA (289 µmol histidine eq/g sample), while trypsin and alcalase hydrolysates significantly had the lower activity (Figure 3c). Hydrolysates prepared using flavourzyme showed the lowest chelation activity (39.2 µmol EDTA eq/g), while trypsin had the highest chelation activity (47.5 µmol EDTA eq/g) (Figure 3d). In general, the antioxidant activity of CGPHs was influenced by the type of enzymes in hydrolysates. Enzyme specificities differences, varying DH, size of molecules, amino acid composition, and sequence could be reason of varying in antioxidant activity observed among difference hydrolysate (Mudgil et al., 2019).

Fractionation of the CGPHs led to improved DPPH RSA and fractioned of all enzymes with lower than 2 kDa (F1) had higher activity than the higher molecular weight. ABTS^+^ RSA was also increased after separation with UF membranes. ABTS^+^ RSA was observed in all fractions. F1 and followed by that F2 fraction showed the higher ABTS^+^ RSA than hydrolysates. F1 fraction of alcalase exhibited the highest activity against ABTS^+^ radical (333.84 µmol trolox eq/g) compared with other fractions (*p* < .05). Similar results were obtained and F1 fraction showed significantly higher (*p* < .05) OH RSA. However, there was no significant difference between trypsin and flavourzyme (Figure 3). The higher content of small peptides in hydrolysates may have higher radical scavenging activity than those with lower contents. This low molecule fractions can easily react with free radical and terminate the radical chain reaction (Agrawal, Joshi, & Gupta, 2019). Similar studies on chickpea protein (Li et al., 2008), corn gluten meal (Zhuang, Tang, & Yuan, 2013), rapeseed protein (He et al., 2013), wheat gluten (Choi, Lim, He, & Hwang, 2019), and perilla seed protein (Kim, Liceaga, & Yoon, 2019) had shown that short peptides are the most efficient antioxidants.

Hydrolysate and F2 fractions exhibited the stronger chelating capacity than F1 (*p* < .05). Decreasing chelating activity may be attributed to the inability of small peptides to form the complex with metals (Noman et al., 2019). Similar results were observed by He et al. (2013) and Tang, Wang, and Yang (2009) on hemp and rapeseed protein hydrolysate. The synergistic effects of higher number of amino acid residues from higher molecular weight of peptides compared with the lower molecular weight peptides could be reason of stronger metal chelating.

Type of amino acid present in hydrolysates also could influence antioxidant activity. The amino acid composition of the F1 of trypsin hydrolysate (the highest DPPH and OH RSA), F1 of alcalase hydrolysate (the highest ABTS^+^ RSA), and F1 of flavourzyme hydrolysate (the highest OH RSA) is described in Table 1. As can be seen, higher RSA of these fractions may be related to high levels of hydrophobic amino acids such as leucine, isoleucine, valine, and proline. Li et al. (2008) and Pownall, Udenigwe, and Aluko (2010) reported similar results with the strongest scavenging activity contained slightly higher amounts of hydrophobic amino acids. The presence of hydrophobic amino acids in peptides increases their solubility in hydrophobic or lipid phase, which simplify the interaction between peptides and donate protons to radical species. Hydrophobic interaction among hydrophobic amino acid residues might enhance the antioxidant activity of peptides (Jin et al., 2016; Ngoh & Gan, 2016; Zambrowicz et al., 2015).

**TABLE 1:** Amino acid profile of protein hydrolysates produced by alcalase, flavourzyme, and trypsin (g/100 g protein)

Amino acid	F1 (alcalase)	F1 (flavourzyme)	F1 (trypsin)	F2 (alcalase)	F2 (trypsin)
Hydrophilic					
Aspartic acid + asparagine	4.69 ± 0.01	6.85 ± 0.22	5.1 ± 0.04	3.21 ± 0.04	2.42 ± 0.09
Glutamic acid + glutamine	22.01 ± 0.05	21.99 ± 0.27	24.08 ± 0.68	29.6 ± 0.92	28.01 ± 0.87
Serine	13.74 ± 0.15	12.05 ± 0.21	11.31 ± 0.09	10.1 ± 0.15	10.65 ± 0.12
Glycine	17.86 ± 0.01	15.23 ± 1.09	15.09 ± 0.09	18.21 ± 0.96	18.19 ± 0.27
Histidine	1.23 ± 0.04	1.27 ± 0.07	0.46 ± 0.06	0.67 ± 0.03	0.25 ± 0.08
Arginine	2.02 ± 0.04	2.78 ± 0.35	2.13 ± 0.04	3.01 ± 0.02	2.51 ± 0.04
Threonine	0.28 ± 0.03	0.55 ± 0.17	0.58 ± 0.05	0.07 ± 0.02	0.68 ± 0.06
Cysteine	0.48 ± 0.02	0.13 ± 0.07	1.2 ± 0.09	0.41 ± 0.02	0.3 ± 0.04
Tyrosine	3.01 ± 0.04	2.57 ± 0.04	2.84 ± 0.1	1.7 ± 0.03	2.61 ± 0.12
Lysine	4.41 ± 0.1	6.02 ± 0.38	5.18 ± 0.65	6.1 ± 0.01	6.53 ± 0.11
	69.73 ± 0.88	69.44 ± 0.71	67.97 ± 0.69	73.08 ± 0.94	72.15 ± 0.74
Hydrophobic					
Alanine	4.89 ± 0.02	3.65 ± 0.15	3.69 ± 0.22	4.04 ± 0.06	4.66 ± 0.16
Proline	5.8 ± 0.01	6.5 ± 0.12	7.06 ± 0.09	7.93 ± 0.07	7.16 ± 0.05
Valine	6.3 ± 0.19	6.45 ± 0.27	4.01 ± 0.05	5.1 ± 0.04	5.48 ± 0.08
Methionine	0.51 ± 0.05	0.69 ± 0.17	0.56 ± 0.07	0.43 ± 0.19	0.47 ± 0.03
Isoleucine	2.21 ± 0.03	3.43 ± 0.21	6.67 ± 0.1	1.39 ± 0.04	2.12 ± 0.09
Leucine	8.4 ± 0.04	8.44 ± 0.29	8.1 ± 0.13	6.01 ± 0.02	6.21 ± 0.08
Phenylalanine	2.16 ± 0.12	1.56 ± 0.09	1.89 ± 0.12	2.03 ± 0.03	1.89 ± 0.03
Tryptophan	–	–	–	–	–
	30.27 ± 0.51	30.72 ± 0.49	32.08 ± 0.3	26.93 ± 1.12	27.99 ± 0.35

The higher Fe^2+^ chelating abilities of peptides from F2 trypsin hydrolysate may return to high content of acidic and basic amino acids of peptides (glutamic acid and glutamine (Glx), aspartic acid, asparagine (Asx), arginine, and lysine) (Table 1). Ambigaipalan et al. (2015) reported that an increased concentration of carboxylic groups and amino groups in branches of the acidic and basic amino acids could enhance metal ion binding and removing metal ions from the system (Ambigaipalan et al., 2015).

### 3.4 α‐Glucosidase inhibition

**FIGURE 4:**
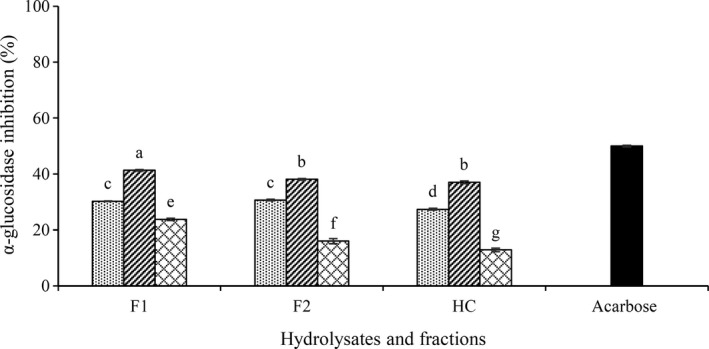
α‐Glucosidase inhibitory activity of CGPH obtained from hydrolysis by different enzymes (alcalase, 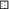 flavourzyme 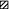, trypsin 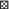) and ultrafiltration fractions. F1, F2, and HC indicate fractions MW < 2, 2–10 kDa, and unfractionated hydrolysate, respectively. Test concentration was 20 mg/ml. Acarbose was used as positive control (IC_50_ = 110 μg/ml). Different letters indicate significant differences among samples (*p* < .05)

As can be seen in Figure 4, all of the hydrolysates from three proteases had a significant difference inhibitory on the α‐glucosidase enzyme. The highest inhibition was related to flavourzyme hydrolysate (37.1%) whereas the lowest inhibitory was obtained by trypsin enzyme (12.8%). This can be due to the specificity of this enzyme and the presence of arginine in the peptide structure. Connolly et al. (2014) reported trypsin hydrolysate of brewers' spent grain protein had the highest amount of inhibition of α‐glucosidase enzyme. As can be expected, the α‐glucosidase inhibitory activity of acarbose (IC_50_ = 110 μg/ml) was higher than CGPH (20 mg/ml). Since synthetic inhibitors are pure compounds but CGPHs are mixtures of proteins and peptides (Connolly et al., 2014).

Ultrafiltration fractions exhibited vary α‐glucosidase inhibitory activities and fractionation improved inhibitory activity. F1 of flavourzyme showed the highest inhibitory effect (41.3%). However, the least inhibition was related to F2 of trypsin (15.9%). These results indicate that short peptides are more potent inhibiting α‐glucosidase activity. Similar results were obtained by Uraipong and Zhao (2018) on rice bran proteins and Mejía, Batista, Fernández, and Fernandes (2019) on beans that reported smaller MW peptides in the digests had greater α‐glucosidase inhibitory activities than the larger peptides.

Inhibitory activity of peptides may be influenced by the composition of the amino acid residues (Di Stefano, Oliviero, & Udenigwe, 2018; Ibrahim, Bester, Neitz, & Gaspar, 2018). The inhibitory peptides are thought to interact more with the active site of α‐glucosidase via hydrogen bonds and electrostatic interactions. Therefore, the presence of amino acids with hydroxyl groups (serine, threonine and tyrosine) or basic amino acids (lysine and arginine) at the amino end of the peptides could play a critical role in alpha‐glucosidase inhibition. Hence, the effect of enzymatic inhibition of fractions in this study could be attributed to the presence of higher amount of amino acids such as serine and lysine (Table 1).

### 3.5 α‐Amylase inhibition

**FIGURE 5:**
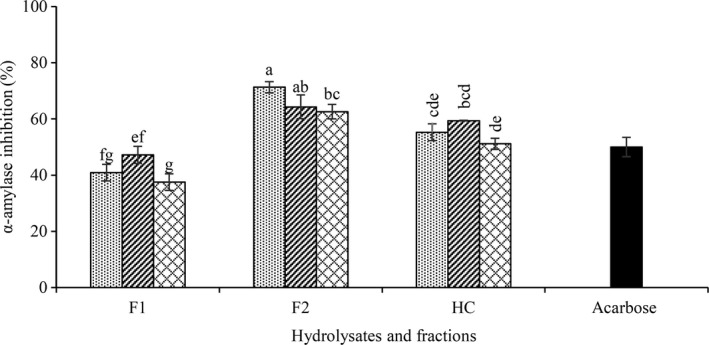
α‐Amylase inhibitory activity of CGPH obtained from hydrolysis by different enzymes (alcalase 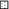, flavourzyme 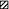, trypsin 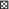) and ultrafiltration fractions. F1, F2, and HC indicate fractions MW < 2, 2–10 kDa, and unfractionated hydrolysate, respectively. Test concentration was 10 mg/ml. Acarbose was used as positive control (IC_50_ = 30 μg/ml). Different letters indicate significant differences among samples (*p* < .05)

One of the effective approaches to control of type 2 diabetes is considered via inhibition of α‐amylase. CGPH obtained by all tree enzymes exhibited the strongest α‐amylase inhibitory activity in which higher than 50% (Figure 5). After ultrafiltration, maximum α‐amylase inhibition in the F2 fraction was observed, while F1 fraction showed the lowest inhibition. F2 fraction of alcalase showed the highest inhibitory effect (71.3%). However, the least inhibition was observed in F1 fraction of trypsin (37.50%). The proposed mechanism of action of the α‐amylase by bioactive peptide was suggested to interact or bind with the enzyme active site and inhibit the interaction between the enzyme and the substrate. Therefore, smaller interaction surface to the substrate occurred and less contact to the multiple‐attack action of amylases occurred (Ngoh & Gan, 2016). Another proposed inhibitory mechanism of biopeptides is the binding of peptides to the allosteric site of the enzyme in the enzyme structure (like calcium and chloride ion site) and create an unstable conformation. This conformation change can restrict the displacement of the enzyme on substrate (Admassu, Gasmalla, Yang, & Zhao, 2018; Siow & Gan, 2016). Calcium ions often have crucial roles in structure, function, and stability of α‐amylases, and the removal of calcium ions from some α‐amylase can inactivate the enzyme (Liao et al., 2019). Amylases are often inhibited by chelating reagents such as EDTA (Hagihara et al., 2001). The strong correlation between the metal ion chelating and α‐amylase inhibitory of this study (*r* = .7) could confirm that the interaction of metal ion chelator peptides with calcium ion in the enzyme structure is one of the reasons for its further decrease in enzyme activity by F2 fraction.

Amino acids found in peptides such as arginine, lysine, aspartic acid + asparagine, glutamic acid + glutamine, proline, leucine, glycine phenylalanine, serine, tryptophan, and tyrosine likely to bind to active domains and have a potential to inhibit the enzyme (Admassu et al., 2018; Ngoh & Gan, 2016; Siow & Gan, 2016). In our study, the higher enzymatic inhibitory of higher molecular weight fractions was probably due to the higher amounts of some amino acids such as proline, glutamic acid + glutamine, aspartic acid + asparagine, leucine, lysine, and glycine.

### 3.6 Dipeptidyl peptidase‐IV (DPP‐IV) inhibition

**FIGURE 6:**
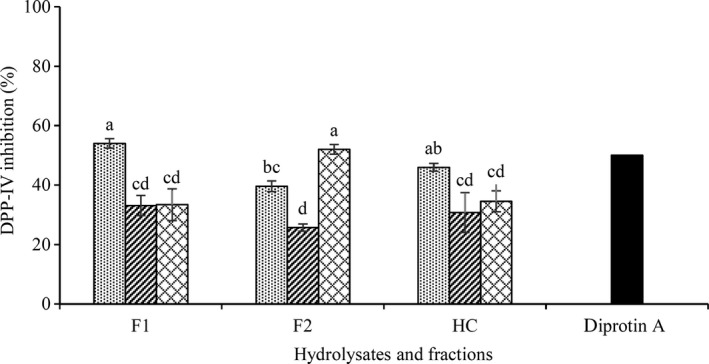
Dipeptidyl peptidase‐IV (DPP‐IV) inhibitory activity of CGPH obtained from hydrolysis by different enzymes (alcalase 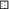, flavourzyme 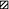, trypsin 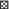) and ultrafiltration fractions. F1, F2, and HC indicate fractions MW < 2, 2–10 kDa and unfractionated hydrolysate, respectively. Test concentration was 5 mg/ml. Diprotin A was used as positive control (IC_50_ = 4 μg/ml). Different letters indicate significant differences among samples (*p* < .05)

Inhibition of DPP‐IV proteinase and enhanced insulin secretion is another approach to lowering postprandial serum glucose. The DPP‐IV inhibitory ability of CGPH and its fractions is shown in Figure 6. Alcalase hydrolysate had the greatest DPP‐IV inhibition (45.9%) while trypsin and flavourzyme hydrolysates showed the lower inhibition (30.7% and 34.5%). Cheung and Li‐Chan (2017) and Mojica and de Mejia (2016) also obtained similar results that inhibition can vary by type of enzymes. In fact, fractions of alcalase and flavourzyme from CGPHs were not significantly higher DPP‐IV inhibitory activity. But the F2 fraction of trypsin hydrolysate showed the stronger inhibition than hydrolysate and F1 fraction. Li‐Chan, Hunag, Jao, Ho, and Hsu (2012) examined inhibitory activity of fractions of hydrolysate obtained from atlantic salmon skin gelatin hydrolyzed by different enzymes and reported that the fraction smaller than 1 kDa had the highest DPP‐IV‐inhibitory activity. Our results (Figure 6) shown that small molecular is not main reason for DPP‐IV inhibition effect and inhibitory activity varied by difference in amino acid composition and length. Similar findings were also reported for hydrolysates generated from Lesser mealworm (Lacroix, Dávalos Terán, Fogliano, & Wichers, 2019) and dairy protein (Lacroix & Li‐Chan, 2012).

## 4 CONCLUSIONS

It was demonstrated that DH and antioxidant and antidiabetic potential of CGPHs can vary depending on the enzymes used. RP‐HPLC chromatograms showed that trypsin hydrolysate had higher levels of high‐hydrophobic peptides, which may be related to its higher antioxidant effect. F1 fraction exhibited highest radical scavenging and α‐glucosidase inhibitory activity. While F2 fraction showed the higher Fe^2+^ chelating and α‐amylase inhibitory activity, hydrolysate and F1 fraction of alcalase and F2 fraction of trypsin showed the highest DPP‐IV inhibitory activity. The amino acid composition of the F1 fractions showed high levels of hydrophobic amino acids such as valine, isoleucine, leucine, and proline. In general, CGPH could be considered as a natural source for ingredients in the functional food and medicinal industries with antioxidant and antidibetic potential.

## CONFLICT OF INTEREST

The authors notify that there are no conflicts of interest.

## ETHICAL APPROVAL

Study's protocols and procedures were ethically reviewed and approved by Tarbiat Modares Research Council.
